# Fas Ligand Gene (Faslg) Plays an Important Role in Nerve Degeneration and Regeneration After Rat Sciatic Nerve Injury

**DOI:** 10.3389/fnmol.2018.00210

**Published:** 2018-06-19

**Authors:** Yuting Li, Yuhua Sun, Min Cai, Huanhuan Zhang, Nannan Gao, Huiwei Huang, Shusen Cui, Dengbing Yao

**Affiliations:** ^1^School of Life Sciences, Co-innovation Center of Neuroregeneration, Jiangsu Key Laboratory of Neuroregeneration, Nantong University, Nantong, China; ^2^Genetic Laboratory, Lianyungang Maternal and Child Health Hospital, Lianyungang, China; ^3^School of Medicine, Nantong University, Nantong, China; ^4^Hand Surgery, China-Japan Union Hospital, Jilin University, Changchun, China

**Keywords:** Wallerian degeneration, sciatic nerve injury, Fas ligand gene (Faslg), rat, nerve regeneration, Schwann cell

## Abstract

Wallerian degeneration (WD) is associated with changes in the expression levels of a large number of genes. However, the effects of these up- or down-regulated genes are poorly understood. We have reported some key factors that are differentially regulated during WD in our previous research. Here, we explored the roles of Fas ligand gene (Faslg) in WD after rat sciatic nerve injury. The data showed that Faslg was up-regulated in injured nerves. Expression changed of Faslg in Schwann cells (SCs) resulted in alterations in the release of related factors. Silencing or overexpression of Faslg affected SC proliferation, migration, and apoptosis through β-catenin, nuclear factor-κB (NF-κB), and caspase-3 pathways *in vivo* and *in vitro*. Our data suggest that Faslg is a key regulatory gene that affects nerve repair and regeneration in peripheral nerve injury. This study sheds new light on the effects of Faslg on peripheral nerve degeneration and/or regeneration.

## Introduction

Nerve injury is one of the most common clinical conditions and may be due to mechanical, chemical, congenital, or pathological causes (Noble et al., 1998; Kouyoumdjian, 2006; Valls-Sole et al., 2011; Zochodne, 2012). Every year, there are more than one million patients with peripheral nerve injury worldwide (Siemionow and Brzezicki, 2009). According to previous studies, injured nerves are capable of regeneration. However, the axon regeneration process is slow and proceeds at a rate of only 1–3 mm/day. As a result, functional recovery is often poor (Sunderland, 1947; Frostick et al., 1998; Scholz et al., 2009). In recent years, the development of nerve transplantation, gene therapy, tissue engineering and cell therapy have led to significant improvements in technologies developed for the repair and regeneration of injured peripheral nerves. Nevertheless, peripheral nerve injury repair remains an active field of study for neuroscientists and medical scholars.

After peripheral nerve injury, distal nerve fibers disconnect from the neuronal cell body, which is a nutrient metabolic center. These fibers then undergo Wallerian degeneration (WD; Idriss and Naismith, 2000). WD, which occurs in both myelin and axons after injury to the peripheral nervous system, is the degeneration of the axons distal to the site of transection (Martini et al., 2013; Chen et al., 2015). WD is essential for nerve repair and regeneration and is an acute pathological response to peripheral nerve injury (Zhao et al., 2010; Dickens et al., 2012; Schleich et al., 2012). However, the molecular mechanisms of WD are not yet completely understood and understanding the genes that regulate WD may reveal mechanisms underlying nerve degeneration and regeneration (Shamash et al., 2002; Vargas and Barres, 2007; Goethals et al., 2010; Tos et al., 2013; Peluffo et al., 2015; Gamage et al., 2017; Sango et al., 2017).

The ability to recover rapidly after peripheral nerve injury is largely due to the apparent plasticity of Schwann cells (SCs), which are peripheral glial cells (Jessen et al., 2015). The latest report in this field indicates that autologous human SCs can be used to support the long-term repair of peripheral nerve injury. In addition, two long-term follow-up surveys have found that SC transplantation for the repair of peripheral nerve injury was initially safe (Gersey et al., 2017). SCs are the main cells that produce myelin. Additionally, their proliferation, migration, and apoptosis play important roles in WD (Jessen et al., 2015; Jessen and Mirsky, 2016). SCs begin to proliferate and migrate to form the Büngner band, which can serve as a guide for growing axons (Chen et al., 2007). The remyelination process begins when SCs contact regenerating axons (Griffin et al., 2010).

In our previous study, we reported that Fas ligand gene (Faslg) is a key regulatory gene of WD and may have a crucial role in nerve injury and repair (Li et al., 2014). Nevertheless, the mechanism underlying the regulation of WD by Faslg remains unknown. Faslg, which is also known as CD95L or tumor necrosis factor superfamily member 6, is a member of the tumor necrosis factor family and is primarily studied as a receptor for Fas (Idriss and Naismith, 2000; Locksley et al., 2001). It encodes a type 2 transmembrane protein that can bind to Fas and induce cell death (Wallach et al., 1999; Locksley et al., 2001; Choi and Benveniste, 2004). The roles of Fas/Fasl in the immune system and in tumor escape are extensively studies. However, its effects are not limited to the immune system, as it also plays important roles in both the reproductive and nervous systems (Suda et al., 1993; Bonetti et al., 2003; Choi and Benveniste, 2004; Preta and Fadeel, 2012). Fas and Fasl are constitutively expressed in the nervous system *in vivo* and *in vitro*, and are both up-regulated after neurological damage (Choi and Benveniste, 2004; Li et al., 2014). Faslg induces secretion of the bioactive nerve growth factor (Mimouni-Rongy et al., 2011) and can lead to the initiation of many intracellular signaling pathways in SCs (Thornhill et al., 2007). Based on the above studies, we hypothesized that Faslg may play important roles in the repair of sciatic nerve injury. We therefore explored the molecular mechanisms underlying the effects of Faslg on sciatic nerve degeneration and regeneration *in vitro* and *in vivo* after injury.

## Materials and Methods

### Rat Model of Sciatic Nerve Injury

We used adult male Sprague-Dawley (SD) rats (180–200 g) to investigate the expression of Faslg during rat sciatic nerve injury and repair. The rats were purchased from the Experimental Animal Center of Nantong University. All animal tests were conducted in accordance with the US National Institutes of Health’s Guide for the Care and Use of Laboratory Animals and by the Key Laboratory of Neuroregeneration Guidelines for the Care and Use of Laboratory Animals. The Institutional Animal Care and Use Committee of Nantong University approved all protocols used in this study.

All animals underwent surgery to establish the sciatic nerve injury model as previously described (Liu et al., 2017). We randomly divided the rats into six groups and performed the operation (Table 1). The rats were anesthetized using complex narcotics (42 mg/kg magnesium sulfate, 17 mg/kg sodium pentobarbital, and 85 mg/kg trichloroacetaldehyde monohydrate). The sciatic nerve was identified and lifted through an incision in the right hind limb. A 1-cm segment was excised after the sciatic nerve was cut. The nerves from one group of rats were immediately used in the assays (0 h). Nerves from the other animals were used 4, 7, 14, 21 and 28 days after surgery (Hirakawa et al., 2003; Gong et al., 2014; Li et al., 2015). Rats in the 0 h group underwent a sham operation.

**Table 1:** Numbers of adult Sprague-Dawley (SD) rats that underwent nerve injury.

Method	Number					
	0 h	4 d	1 w	2 w	3 w	4 w
Real-time PCR	3	3	3	3	3	3
Western blot	6	6	6	6	6	6
Immunohistochemistry	3	3	3	3	3	3
Sum	216					

*The assays were repeated three times. Abbreviations: w, weeks; PCR, polymerase chain reaction*.

### SC Primary Culture

We prepared SCs using the protocol of Weinstein and Wu (2001). Briefly, 0–3 day-old SD rat sciatic nerves were dissected and minced (Table 2), incubated at 37°C in 3 mg/ml collagenase for 30–40 min, and trypsinized at 37°C for 8–10 min. The cells were then cultured at 37°C and 5% CO_2_ (Thermo Scientific Forma CO_2_ incubator) in plastic plates coated with poly-L-lysine containing Dulbecco’s modified Eagle’s medium and 10% fetal bovine serum. of the primary SC cultures were treated with cytosine arabinoside at 10 μM. The surviving fibroblasts were then eliminated using polyclonal anti-Thy1.1 antiserum (Sigma; St. Louis, MO, USA) and rabbit complement (Invitrogen; Carlsbad, CA)-mediated lysis. The SC culture obtained using this procedure was >95% pure, as confirmed using S100B and Hochest 33342 immunocytochemistry (ICC).

**Table 2:** Numbers of neonatal SD rats used.

Method	Number	
	Faslg knockdown	Faslg overexpression
Immunohistochemistry	14	
Transfection validation (real-time PCR)	7	7
Transfection validation (Western Blot)	7	7
Annexin V-FITC apoptosis detection	7	7
Cell-Light™ EdU DNA cell proliferation	7	7
Transwell cell migration	7	7
Analysis of related factors (real-time PCR)	7	7
Analysis of related proteins (Western Blot)	7	7
Sum	336	

*The assays were repeated three times. Abbreviations: Faslg, Fas ligand gene; PCR, polymerase chain reaction; FITC, fluorescein isothiocyanate; EdU, 5-Ethynyl-2′-deoxyuridine*.

### Faslg Small Interfering RNA (siRNA) Transfection in SCs

We used three different Faslg small interfering RNAs (siRNAs; Table 3) for siRNA interference analysis. Faslg siRNAs (Integrated Biotech Solutions; Shanghai, China) were transfected into purified SCs. Lipofectamine RNAi MAX transfection reagent (Invitrogen) was used according to the manufacturer’s instructions. Negative control (NC) siRNA and a blank control that raised normally were also tested. We repeated the above experiments three times for data analysis.

**Table 3:** Faslg small interfering RNA primers.

Gene	Sequence
siRNA-1	F: 5′ CUCUAAAGAAGAAGGACAACA 3′
	R: 5′ UUGUCCUUCUUCUUUAGAGGG 3′
siRNA-2	F: 5′ GGAACUGUCUGAUGUUAAAUG 3′
	R: 5′ UUUAACAUCAGACAGUUCCUU 3′
siRNA-3	F: 5′ CCAUUUAUAUGUCAACAUAUC 3′
	R: 5′ UAUGUUGACAUAUAAAUGGUC 3′
NC	F: 5′ UUCUCCGAACGUGUCACGUTT 3′
	R: 5′ ACGUGACACGUUCGGAGAATT 3′

*Abbreviations: siRNA, small interfering RNA; F, forward; R, reverse; NC, negative control*.

### Overexpression of Faslg in SCs

To examine the effects of Faslg overexpression, we transfected the SCs with a mixture of X-treme GENE HP DNA Transfection Reagent (Roche, Germany) and pcDNA3.1(+)-Faslg plasmid, or a mixture of X-treme GENE HP DNA Transfection Reagent and an empty vector for 48 h. We performed real-time quantitative polymerase chain reaction (qPCR) and Western blot analyses. The above experiments were all repeated three times.

### Immunohistochemistry Analysis of Faslg Expression

We prepared distal sciatic nerve samples, fixed them in 4% paraformaldehyde, and dehydrated them in 30% sucrose solution. The distal sciatic nerve sections were into 12-μm-thick sections using a Leica CM3050 S Research Cryostat Microtome and mounted onto slides. We then rinsed the nerve sections in phosphate-buffered saline and permeabilized them in a solution of 5% goat serum, 0.3% Triton X-100 and 1% bovine serum albumin in phosphate-buffered saline prior to staining them. The nerve sections were incubated with a mouse monoclonal anti-Faslg (1:50, Santa Cruz, USA) antibody and a mouse monoclonal anti-S100 (1:500, Sigma) antibody for 12 h at 4°C. and the sections were then incubated for 2 h with goat anti-rabbit immunoglobulin G Alexa Fluor 488 (1:400, Invitrogen) and goat anti-mouse IgG Cy3 (1:400, Sigma) at room temperature. Hoechst 33342 was used to counterstain the nerve sections for 10 min. We observed all of the samples and acquired images using a laser microscope (FV10i-oil; Tokyo, Japan).

### Real-Time qPCR Analysis

To investigate the expression level of Faslg, total RNA was extracted using Trizol extraction reagent (Qiagen; CA, USA) and cDNA was synthesized using a cDNA Reverse Transcription Kit (Qiagen) according to the manufacturer’s protocol (Implen NanoPhotometer; Applied Biosystems^®^ 2720 Thermal Cycler). We performed real-time qPCR using a PCR system according to the manufacturer’s instructions (Applied Biosystems^®^ StepOnePlus™ Real-Time PCR System). The reactions were carried out in triplicate. We repeated these experiments three times and used the comparative cycle threshold method for analysis. The forward and reverse PCR primers used are presented in Table 4.

**Table 4:** Real-time quantitative PCR primers.

Gene	Sequence
Faslg	F: 5′ CACCAACCACAGCCTTAGAGTATCA 3′
NM_012908.1	R: 5′ ACTCCAGAGATCAAAGCAGTTCCA 3′
bcl2	F: 5′ GCAGAGATGTCCAGTCAGC 3′
NM_016993.1	R: 5′ CCCACCGAACTCAAAGAAGG 3′
bax	F: 5′ TGCAGAGGATGATTGCTGAC 3′
NM_017059.2	R: 5′ GATCAGCTCGGGCACTTTAG 3′
bFGF	F: 5′ CCCGCACCCTATCCCTTCACAGC 3′
NM_019305.2	R: 5′ CACAACGACCAGCCTTCCACCCAAA 3′
NT3	F: 5′ GACAAGTCCTCAGCCATTGACATTC 3′
NM_001270870.1	R: 5′ CTGGCTTCTTTACACCTCGTTTCAT 3′
Nf2	F: 5′ CTGGGATTGGGTTCATGGGTGGAT 3′
NM_013193.1	R: 5′ AGGAAGCCCGAGAAGCAGAGCG 3′
PKCα	F: 5′ GAACACATGATGGACGGGGTCACGAC 3′
NM_001105713.1	R: 5′ CGCTTGGCAGGGTGTTTGGTCATA 3′
GAPDH	F: 5′ TGGAGTCTACTGGCGTCTT 3′
NM_017008.4	R: 5′ TGTCATATTTCTCGTGGTTCA 3′

*Abbreviations: Faslg, Fas ligand gene; F, forward; R, reverse; bcl2, B-cell lymphoma 2; bax, Bcl-2-associated X protein; bFGF, basic fibroblast growth factor; NT3, neurotrophin-3; Nf2, neurofibromin 2; PKCα, protein kinase C α; GAPDH, glyceraldehyde 3-phosphate dehydrogenase*.

### Western Blot Analysis of Faslg Expression

We extracted proteins from injured nerve samples and SCs using protein lysis buffer containing protease inhibitors. We separated equal amounts of protein using sodium dodecyl sulfate polyacrylamide gel electrophoresis and then transferred the separated proteins to polyvinylidene fluoride membranes (Bio-Rad Protein Gel Electrophoresis Chamber System). The membranes were blocked using non-fat milk in Tris-buffered saline containing Tween-20 and incubated with primary antibodies. To normalize the total levels of proteins, β-actin was used as a reference in our analyses. The experiments were repeated three times.

### Cell Proliferation Assay

To assess SC proliferation, 2 × 10^5^ cells/mL were plated onto 0.01% poly-L-lysine-coated plates. Cell proliferation was assessed after the SCs were transfected for 2 days. 5-Ethynyl-2′-deoxyuridine (EdU) was added to the cell culture for 2 h. The SCs were then fixed using formaldehyde. A Cell-Light EdU DNA Cell Proliferation Kit was used to measure cell proliferation according to the manufacturer’s protocol. We determined the ratio of EdU-positive cells using images of randomly selected fields under a DMR fluorescence microscope (Leica DMI 4000B Research Inverted Microscope). The assays were performed three times.

### Cell Migration Assay

We used 6.5-mm transwell chambers with 8-μm pores to examine SC migration, as described previously (Liu et al., 2017). SCs were resuspended in Dulbecco’s modified Eagle’s medium and transferred to the top chamber. The SCs were then allowed to migrate into the lower chamber. The SCs were stained with Cresyl Violet. We imaged and counted the cells using a DMR inverted microscope. The assay was performed three times.

### Flow Cytometry

We assessed SC apoptosis using the Annexin V-FITC Apoptosis detection kit according to the manufacturer’s instructions. SCs were collected for flow cytometry analysis and labeled with fluorescein isothiocyanate and annexin V in binding buffer. They were then incubated in propidium iodide on ice in the dark. The numbers of apoptotic cells were measured using FACScan flow cytometry (BD FACSCalibur™ Flow Cytometer). The assay was performed three times.

### *In Vivo* Assay

It has been reported that Chimeric Rabies Virus Glycoprotein Fragment (RVG-9R; AnaSpec, Belgium) may be used as a tool for the delivery of siRNA to the nervous system (Kumar et al., 2007; Rassu et al., 2017). The Sequence of RVG-9R is H-Tyr-Thr-Ile-Trp-Met-Pro-Glu-Asn-Pro-Arg-Pro-Gly-Thr-Pro-Cys-Asp-Ile-Phe-Thr-Asn-Ser-Arg-Gly-Lys-Arg-Ala-Ser-Asn-Gly-Gly-Gly-Gly-Arg-Arg-Arg-Arg-Arg-Arg- Arg-Arg-Arg-OH (3-letter code; YTIWMPENPRPG-TPCD IFTNSRGKRASNGGGGRRRRRRRRR [1-letter code]). The sciatic nerves of adult male SD rats were exposed and cut to create a 1-cm gap in the left hind limb. A 1.0-mm silicone tube was implanted to bridge the nerve gap. We randomly divided the rats into four groups (Table 5): a complex of RVG-9R and Faslg siRNA was injected into the tube in the experimental group and the control group. The Faslg plasmid (pcDNA3.1(+)-Faslg) and Matrigel (BD BioCoat, USA) were used to induce Faslg overexpression *in vivo*. A complex of pcDNA3.1(+)-Faslg and Matrigel also injected into the tube similar to Faslg siRNA. We euthanized the rats 7 and 14 days after surgery and collected the silicone tubes. We then performed real-time qPCR and Western blot analyses. We analyzed the nerve samples three times.

**Table 5:** Numbers of adult SD rats used in the *in vivo* assay.

Method	Number							
	pcDNA3.1(+)-7 d	pcDNA3.1(+)-Faslg-7 d	pcDNA3.1(+)-14 d	pcDNA3.1(+)-Faslg-14 d	NC-7 d	siRNA-Faslg-7 d	NC-14 d	siRNA-Faslg-14 d
Real-time PCR	3	3	3	3	3	3	3	3
Western blot	6	6	6	6	6	6	6	6
Immunohistochemistry	3	3	3	3	3	3	3	3
Sum	288							

*The experiments were repeated three times. Abbreviations: NC, negative control; siRNA, short interfering RNA; Faslg, Fas ligand gene; PCR, polymerase chain reaction*.

### Assessment of Apoptosis Using Terminal Deoxynucleotidyl Transferase-Mediated Nick-End Labeling (TUNEL)

We performed terminal deoxynucleotidyl transferase-mediated nick-end labeling (TUNEL) of the frozen slices (Promega, USA). After TUNEL, the nuclei were labeled using Hochest 33342, and TUNEL-positive cells were observed using a Leica Imager and an M2 fluorescence microscope (Leica) with an objective. The average number of apoptotic cells was calculated averaging the numbers of TUNEL-positive apoptotic cells in each sample.

### Statistical Analysis

We performed statistical analysis using SPSS 15.0 for Windows (SPSS, IL, USA). The Shapiro-Wilk test was used to assess normality. Student’s *t* tests were used for comparisons between two groups. Group differences were analyzed using one-way analyses of variance or Mann-Whitney U tests, as appropriate. *P*-values less than 0.05 were considered statistically significant. All data are expressed as mean ± standard deviation.

## Results

### Faslg Expression in Injured Sciatic Nerves and SCs

**Figure 1:**
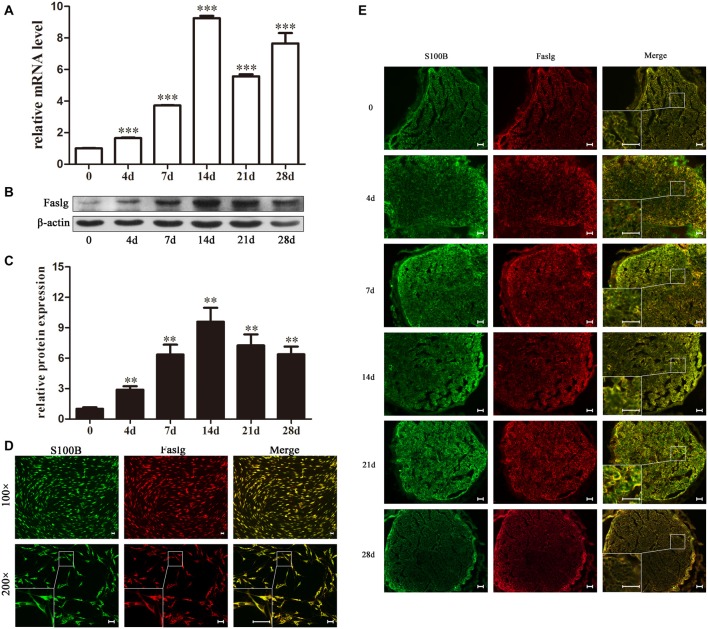
Fas ligand gene (Faslg) expression in the injured rat sciatic nerve and cultured Schwann cells (SCs). **(A)** Real-time quantitative polymerase chain reaction (qPCR) analysis of Faslg expression in injured sciatic nerves 0, 4, 7, 14, 21 and 28 days post-injury. Glyceraldehyde 3-phosphate dehydrogenase (GAPDH) expression was used to normalize the data. The average of three independent experiments is shown as mean ± standard error of the mean (SEM; ***p < 0.001). **(B)** Western blot analysis of Faslg expression in injured sciatic nerves 0, 4, 7, 14, 21 and 28 days post-injury. β-actin levels were used as control. **(C)** Relative protein expression levels of Faslg as determined by the Western blot analysis (**p < 0.01). **(D)** Immunofluorescence staining of S100B (green) and Faslg (red) and the overlay of the two signals in cultured SCs (scale bar, 50 μm). **(E)** Immunofluorescence staining of S100B and Faslg and the overlay of the two signals in injured sciatic nerve stumps. S100B was used as an SC-specific marker (scale bar, 50 μm). Each experiment was repeated three times. Bar = 50 μm.

In this study, we used real-time qPCR and Western blot to determine the expression levels of Faslg 4, 7, 14, 21 and 28 days after sciatic nerve injury. The real-time qPCR results indicated that Faslg expression was remarkably increased after injury. The Western blotting results were consistent with the gene expression results. Glyceraldehyde 3-phosphate dehydrogenase (GAPDH) levels were used as the gene expression control and β-actin levels were used as the protein expression control (Figure 1). Immunohistochemisty was used to visualize the localization of Faslg and S100B within the sciatic nerve and in the cultured SCs at different time points after the injury. The SCs were immunostained using anti-S100B. Faslg and S100B were colocalized in SCs, indicating that Faslg is expressed in SCss in the rat sciatic nerve. Faslg was also present in cultured SCs (*p < 0.05; Figure 1). All data were assessed using analysis of variance and Scheffé’s *post hoc* tests (*p < 0.05).

### Faslg Knockdown and Overexpression Affected Gene Expressions in SCs *in Vitro*

**Figure 2:**
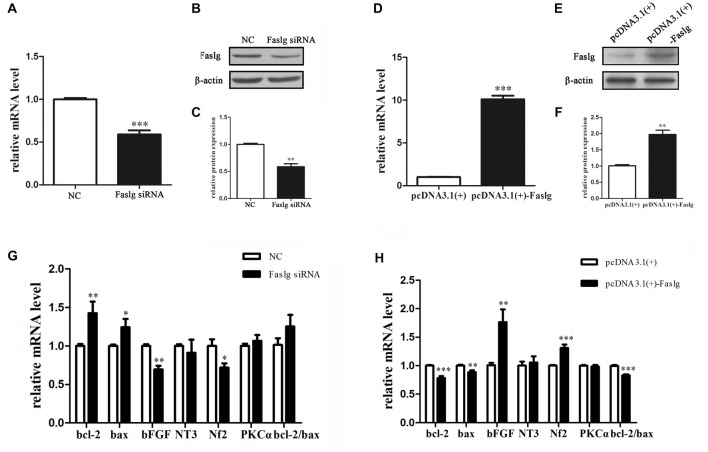
Expression changes following Faslg knockdown or overexpression SCs. **(A,B,C)** Faslg in SCs was down-regulated following transfection with Faslg small interfering RNA (siRNA) when compared to siRNA control (NC). **(D,E,F)** Faslg in SCs was up-regulated following transfection with the pcDNA3.1(+)-Faslg plasmid when compared to control (pcDNA3.1(+)). **(G)** Real-time qPCR analysis of B-cell lymphoma-2 (bcl-2), Bcl-associated X protein (bax), basic fibroblast growth factor (bFGF), Neurotrophin 3 (NT3), neurofibromin 2 (Nf2) and Protein kinase C α (PKCα) mRNA expression levels after Faslg siRNA transfection in SCs. GAPDH was used to normalize values to those of the negative control (NC). **(H)** Real-time qPCR analysis of bcl-2, bax, bFGF, NT3, Nf2 and PKCα mRNA expression levels after pcDNA3.1-Faslg plasmid transfection in SCs, with GAPDH used to normalize values to those of the NC. Independent-samples *t* tests were used. The average of three independent experiments is shown as mean ± SEM (*p < 0.05, **p < 0.01, ***p < 0.001).

We synthesized three specific Faslg siRNAs. One of the three siRNAs was found to notably reduce Faslg mRNA expression levels (Figure 2). We used this siRNA in later experiments. To investigate the potential functions of Faslg in SCs, we also assessed the gene expression levels of several related factors, including Bcl-2-associated X protein (Bax) and the pro-apoptotic factors B-cell lymphoma 2 (Bcl-2), neurofibromin 2 (Nf2), neurotrophin 3 (NT3), protein kinase C α (PKCα) and basic fibroblast growth factor (bFGF) after Faslg knockdown or overexpression in transfected SCs. The real-time qPCR results showed that bFGF and Nf2 mRNA expression levels were down-regulated in SCs following Faslg knock-down and up-regulated following Faslg overexpression. In contrast, the expression levels of Bcl-2 and Bax mRNA were up-regulated following Faslg knockdown and down-regulated following Faslg overexpression in SCs (Figure 2; *p < 0.05). Therefore, the expression changes of Faslg altered gene expression levels in cultured SCs *in vitro*.

### Faslg Affects SC Proliferation, Migration and Apoptosis *in Vitro*

**Figure 3:**
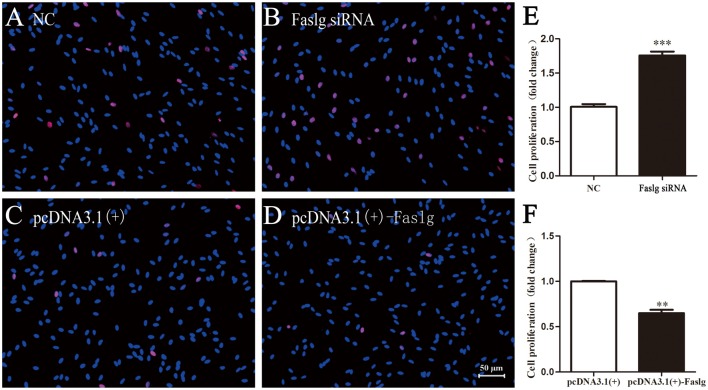
Faslg knockdown and overexpression affected SC proliferation. **(A,B)** Silencing of Faslg by transfection with Faslg siRNA significantly induced SC proliferation when compared to the NCs. **(C,D)** Overexpression of Faslg following transfection with the pcDNA3.1(+)-Faslg plasmid significantly inhibited SC proliferation when compared to the NCs. **(E)** Relative numbers of SCs following Faslg knockdown. **(F)** Relative numbers of SCs following overexpression of Faslg (scale bar, 50 μm). Independent-samples *t* tests were used. The average of three independent experiments is shown as mean ± SEM (**p < 0.01, ***p < 0.001).

**Figure 4:**
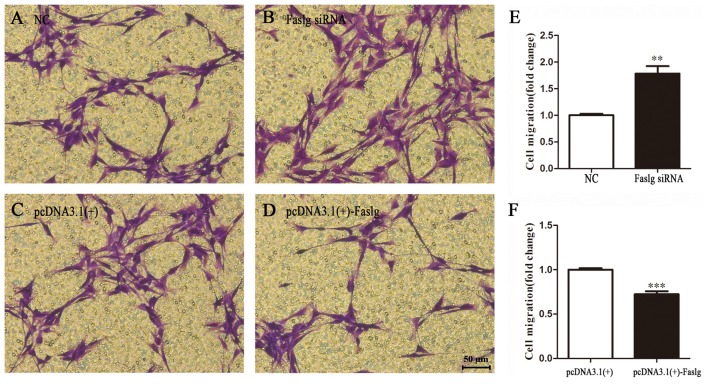
Faslg knockdown and overexpression affect SC migration. **(A,B)** Silencing of Faslg following transfection with Faslg siRNA in SCs significantly induced SC migration when compared to (NCs). **(C,D)** Overexpression of Faslg following transfection with the pcDNA3.1(+)-Faslg plasmid significantly inhibited SC migration when compared to the NCs. **(E)** Relative numbers of SCs following Faslg knockdown. **(F)** Relative numbers of SCs following overexpression of Faslg. Independent-samples *t* tests were used. The average of three independent experiments is shown as mean ± SEM (**p < 0.01, ***p < 0.001).

**Figure 5:**
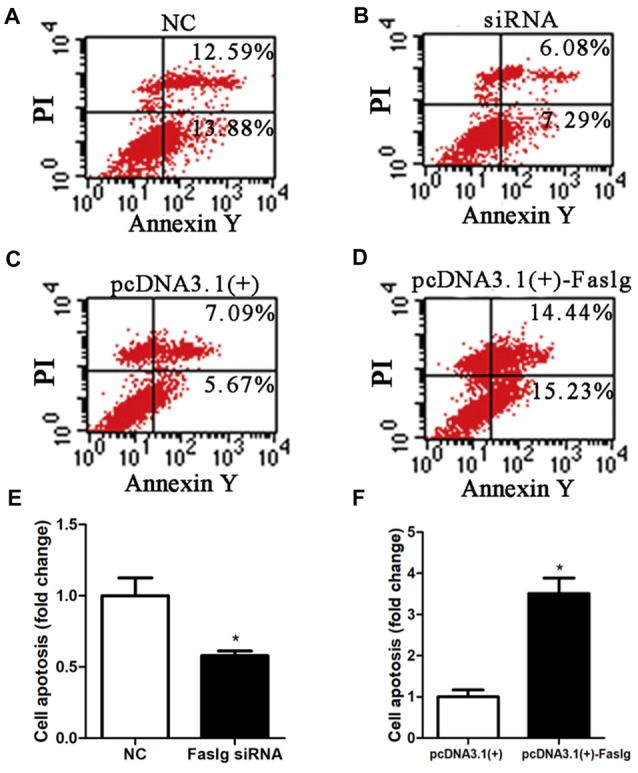
Faslg knockdown and overexpression affected SC apoptosis. **(A,B)** Silencing Faslg following transfection with Faslg siRNA significantly inhibited apoptosis in SCs when compared to the NCs. **(C,D)** Overexpression of Faslg in SCs following transfection with the pcDNA3.1(+)-Faslg plasmid significantly induced SC apoptosis when compared to the NCs. **(E)** Relative numbers of SCs following Faslg knockdown. **(F)** Relative numbers of SCs following overexpression of Faslg. Independent-samples *t* tests were used. The average of three independent experiments is shown as mean ± SEM (*p < 0.05).

To determine the functions of Faslg in SCs, primary SCs were transfected with Faslg siRNA, pcDNA3.1(+)-Faslg plasmid, or NC vector, and the effects of these transfections on cell proliferation, migration and apoptosis were examined *in vitro*. An EdU-based assay indicated that SCs transfected with Faslg siRNA had increased proliferation, while those transfected with pcDNA3.1(+)-Faslg had decreased proliferation (Figure 3). SCs transfected with Faslg siRNA had increased cell migration, while those transfected with pcDNA3.1(+)-Faslg plasmid had decreased cell migration when compared to the control cells (Figure 4). The apoptosis rate was decreased in SCs transfected with Faslg siRNA and increased in SCs transfected with pcDNA3.1(+)-Faslg when to the control cells (Figure 5). These results indicated that silencing Faslg induced SC proliferation and migration, while enhancing Faslg expression reduced SC apoptosis. Briefly, Faslg affects SC proliferation, migration and apoptosis *in vitro*.

### Faslg Affects the β-Catenin, Nuclear Factor-κB (NF-κB) and Caspase-3 Pathways *in Vitro*

**Figure 6:**
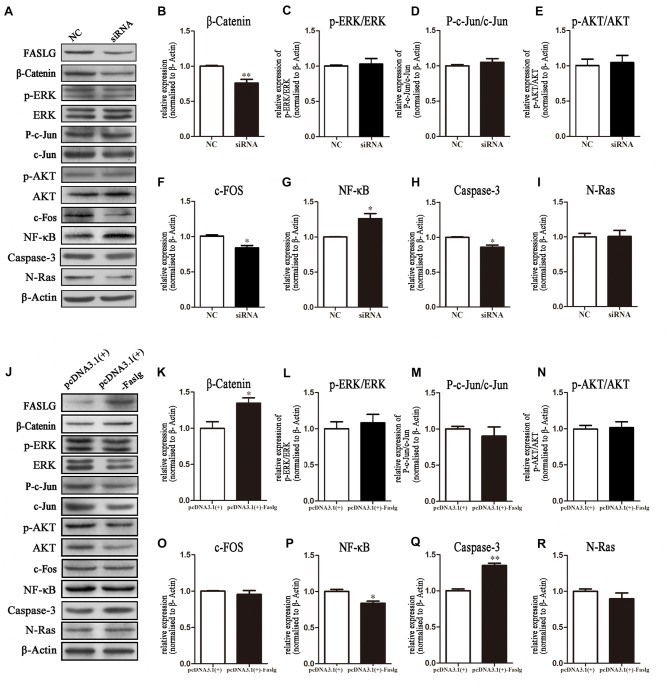
Expression levels of β-Catenin, p-extracellular regulated protein kinase (ERK)/ERK, p-AKT/AKT, nuclear factor-κB (NF-κB) and caspase-3 following Faslg knockdown or overexpression in SCs. **(A)** Western blot analyses of β-catenin, p-ERK/ERK, p-AKT/AKT, p-c-Jun/c-Jun, c-Fos, NF-κB, caspase-3 and N-Ras expression after Faslg siRNA transfection in SCs, with β-actin used to normalize the levels to those of NCs. **(B–I)** Relative protein levels of β-catenin, p-ERK/ERK, p-AKT/AKT, p-c-Jun/c-Jun, c-Fos, NF-κB, caspase-3 and N-Ras, as determined by Western blot analysis. **(J)** Western blot analyses of β-catenin, p-ERK/ERK, p-AKT/AKT, p-c-Jun/c-Jun, c-Fos, NF-κB, caspase-3 and N-Ras expression after transfection with the pcDNA3.1(+)-Faslg plasmid in SCs, with β-actin used to normalize the levels to those of NCs. The average of three independent experiments is shown as mean ± SEM. **(K–R)** Relative protein levels of β-Catenin, p-ERK/ERK, p-AKT/AKT, p-c-Jun/c-Jun, c-Fos, NF-κB, caspase-3 and N-Ras, as determined by Western blot analysis. The average of three independent experiments is shown as mean ± SEM (*p < 0.05, **p < 0.01). Independent-samples *t* tests were used.

The effects of Faslg on gene expression in SCs indicated that altered Faslg expression could affect the release of related factors. We examined whether Faslg affected cell signaling pathways *in vitro* using cultured SCs. We measured the protein expression levels of β-catenin, phosphorylated extracellular regulated protein kinase (p-ERK)/ERK, p-AKT/AKT, p-c-Jun/c-Jun, c-Fos, nuclear factor-κB (NF-κB), caspase-3 and neuroblastoma Ras (N-Ras) and compared them to those of the NC. The levels of β-catenin, NF-κB and caspase-3 were significantly altered after Faslg siRNA or pcDNA3.1(+)-Faslg plasmid transfection (*p < 0.05, **p < 0.01). Interestingly, the expression of c-Fos was significantly altered when Faslg was silenced in SCs, but not with SC overexpression. These results indicate that the β-catenin, NF-κB, c-Fos and caspase-3 signaling pathways can be activated by Faslg (Figure 6). Therefore, Faslg may play an important role in regulating the β-catenin, NF-κB, c-Fos and caspase-3 signaling pathways in cultured SCs *in vitro*.

### Altered Faslg Expression Affects Nerve Degeneration and Regeneration *in Vivo*

**Figure 7:**
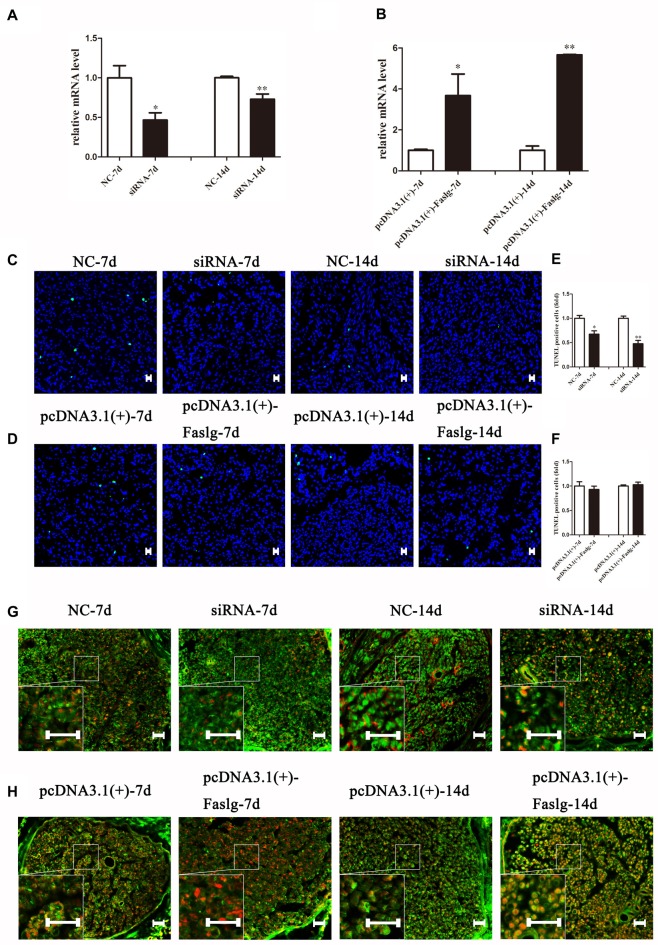
The effect of Faslg in the injured sciatic nerve after knockdown or overexpression. **(A)** Faslg expression in the injured sciatic nerve was down-regulated following transfection with Faslg siRNA when compared to siRNA control (NC). **(B)** Faslg expression in the injured sciatic nerve was up-regulated following transfection with the pcDNA3.1(+)-Faslg plasmid when compared to control (pcDNA3.1(+)). **(C)** Apoptosis assay in the injured sciatic nerve after transfection with Faslg siRNA, as determined using terminal deoxynucleotidyl transferase-mediated nick-end labeling (TUNEL) staining. **(D)** Apoptosis assay in the injured sciatic nerve after transfection with the pcDNA3.1(+)-Faslg plasmid, as determined using TUNEL staining. **(E)** Relative numbers of apoptotic cells in the injured sciatic nerve following Faslg knockdown. **(F)** Relative numbers of apoptotic cells in the injured sciatic nerve following overexpression of Faslg. The average of three independent experiments is show as mean ± SEM (*p < 0.05, **p < 0.01). Independent-samples *t* tests were used. **(G)** Immunofluorescence staining showing the overlay (yellow) of S100B (green) and FasL (red) signals in the distal sciatic nerve stumps following Faslg knockdown or control treatment in rats at the indicated times. **(H)** Immunofluorescence staining showing the overlay (yellow) of the S100B (green) and FasL (red) signals in the distal sciatic nerve stumps following overexpression of Faslg or control treatment in rats at the indicated times. Bar = 50 μm.

**Figure 8:**
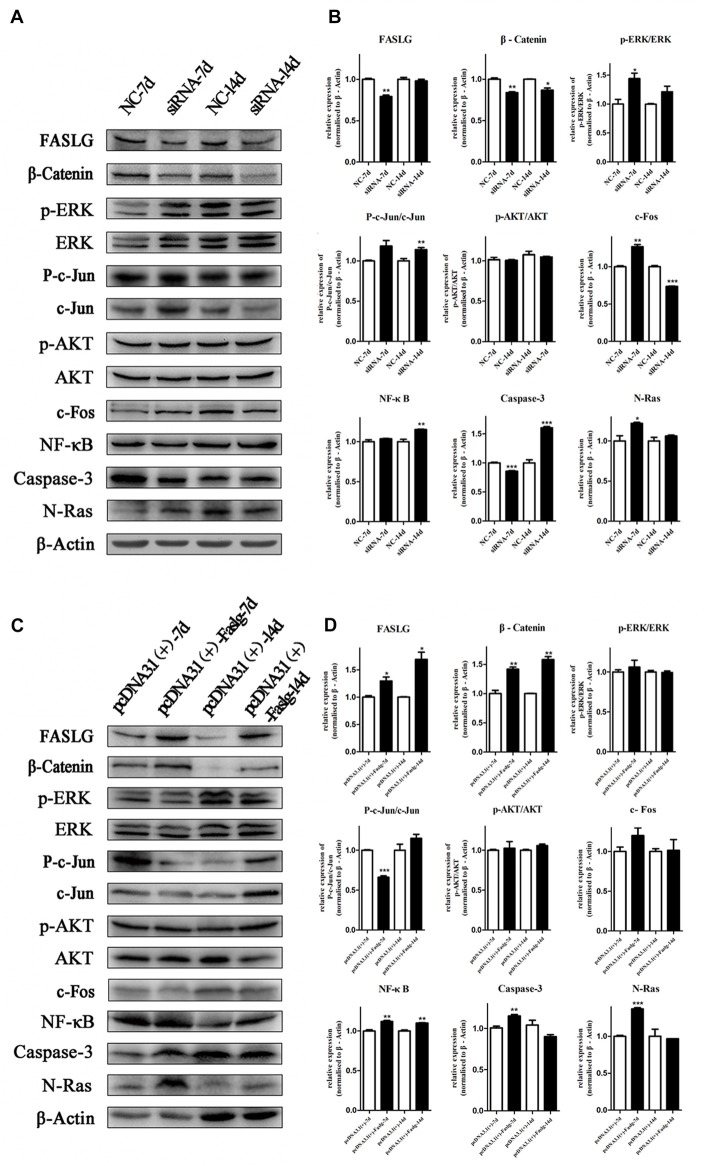
Altered Faslg expression affects the β-catenin, NF-κB and caspase-3 pathways *in vivo*. **(A)** Western blot analyses of Faslg, β-Catenin, p-ERK/ERK, p-AKT/AKT, p-c-Jun/c-Jun, c-Fos, NF-κB, caspase-3 and N-Ras expression levels after exposure of the injured rat sciatic nerve to Faslg siRNA for 7 and 14 days. β-actin levels were used as control. **(B)** Relative protein expression levels of Faslg, β-Catenin, p-ERK/ERK, p-AKT/AKT, p-c-Jun/c-Jun, c-Fos, NF-κB, caspase-3 and N-Ras, as determined using Western blot analysis. **(C)** Western blot analyses of Faslg, β-Catenin, p-ERK/ERK, p-AKT/AKT, p-c-Jun/c-Jun, c-Fos, NF-κB, caspase-3 and N-Ras expression after exposure of the injured rat sciatic nerve to the pcDNA3.1(+)-Faslg plasmid for 7 and 14 days, with β-actin used to normalize the levels to those of NCs. **(D)** Relative protein expression levels of Faslg, β-catenin, p-ERK/ERK, p-AKT/AKT, p-c-Jun/c-Jun, c-Fos, NF-κB, caspase-3 and N-Ras, as determined using Western blot analysis. The average of three independent experiments is shown as mean ± SEM (*p < 0.05, **p < 0.01, ***p < 0.001). Independent-samples *t* tests were used.

To determine the effects of Faslg on nerve degeneration and regeneration after sciatic nerve injury *in vivo*, we analyzed the effects of Faslg on sciatic nerve repair and regeneration 7 and 14 days after injury in the rats. After the injured sciatic nerve was exposed to Faslg siRNA, pcDNA3.1(+)-Faslg, or NC for 7 or 14 days, we performed real-time qPCR, TUNEL, immunohistochemistry, and Western blot analysis. Real-time qPCR revealed that Faslg siRNA and pcDNA3.1(+)-Faslg had specific effects *in vivo* (Figure 7). We thus explored the apoptotic effects of Faslg *in vivo* using TUNEL. The numbers of apoptotic cells were significantly reduced when Faslg expression was silenced, while the numbers of apoptotic cells were almost unchanged when Faslg was overexpressed (Figure 7). We used immunohistochemistry to study morphological changes *in vivo*. The expression of Faslg led to changes in morphology (Figure 7). This result was consistent with the real-time qPCR data. To explore whether Faslg affected the above-mentioned signaling pathways *in vivo*, we performed Western blots and discovered that the β-catenin, NF-κB, p-c-Jun/c-Jun, N-Ras and caspase-3 signaling pathways underwent had undergone stable changes following Faslg silencing or overexpression (Figure 8). The p-ERK/ERK and c-Fos signaling pathways also underwent changes following Faslg interference or overexpression (Figure 8). These results indicated that Faslg plays an important role in degeneration and regeneration in the sciatic nerve after injury *in vivo*.

## Discussion

The nervous system consists of the central nervous system (CNS) and the peripheral nervous system. The biggest difference between the two systems is that the peripheral nerve had an amazing ability to regenerate. After injury to a peripheral nerve, the distal end of the nerve will undergo WD. WD is the transient and acute pathological response to peripheral nerve injury (Martini et al., 2013; Wu et al., 2013; Jung et al., 2014; Peluffo et al., 2015). Rapid developments in the field of neuroscience have led to the study of the molecular mechanism underlying many important processes (Gong et al., 2014; Li et al., 2015). We have used DNA microarray technology to detect changes in mRNA expression both in the early and late stages of WD and in the sciatic nerve of adult rats after transection. Our findings indicate that after nerve injury, Faslg expression peaks on day 7 and reaches maximum levels on day 14 (Yao et al., 2012, 2013; Pan et al., 2017). Interestingly, day 7 is a key time point during WD because axon regrowth begins 7 days after nerve injury (Mietto et al., 2015).

Related studies have shown that Faslg not only plays a pivotal role in the immune system, but also has an equally important role in the nervous system and may also contribute to nerve degeneration and regeneration (Mimouni-Rongy et al., 2011). In 2014, it was reported that Faslg is involved in regulation of the core position during sciatic nerve injury repair and regeneration in rats (Li et al., 2014), and role in inducing nerve regeneration was specifically proven. However, the specific mechanism underlying the effects of Faslg has not been specifically studied. Elucidation of this mechanism was the main purpose of this study. This study was divided into three parts: verification of the expression of Faslg *in vivo*, exploration of the function and mechanism of Faslg action *in vitro*, and study of the effect of Faslg on degeneration and regeneration in sciatic nerve injury *in vivo*.

Our research group has previously reported that the expression of Faslg at the gene and protein levels is significantly increased during WD after sciatic nerve injury (Li et al., 2014). It may thus be speculated that Faslg may play an important role in SC behavior. Our results indicate that Faslg could inhibit SC proliferation and migration, although it also significantly promoted apoptosis in SCs *in vitro* in this study. *In vivo*, we found that Faslg could indeed have an effect on SCs apoptosis, a result that was consistent with those of the *in vitro* study. The effects of different factors in a cell are not isolated. Instead, the different factors interact with each other and are related to underlying molecular mechanisms. It has been reported that Bcl-2 is a member of the anti-apoptotic family and that Bax binds to Bcl-2 (Oltvai et al., 1993). bFGF has also been reported to inhibit neuronal apoptosis and exhibits protective neuropathic effects (Zhang et al., 2013). We measured the expression levels of genes such as bcl-2, bax, bFGF, NT3, Nf2 and PKCα following Faslg siRNA treatment or overexpression in SCs. Our results indicate that the effects of Faslg on SCs might be regulated by Bcl-2, Bax, bFGF and Nf2. These factors have also been reported to interact with important factors in the repair of sciatic nerve injury, such as transforming growth factor-β1 and secreted phosphoprotein 1 (Li et al., 2015; Liu et al., 2017).

It has been reported that the Fas/FasL complex can promote the migration and proliferation of endothelial cells through the Fas-associated protein with death domain- FADD-like IL-1β-converting enzyme inhibitory protein-tumor necrosis factor receptor associated factor-NF-κB pathway (Zhang et al., 2015). NF-κB can also directly activate Fas ligand expression to induce Fas cascade death (Kucharczak et al., 2003). At the same time, NF-κB transcription factors may also inhibit apoptosis by modulating the Bcl-extra large anti-apoptotic factor (Chen et al., 2000). Although c-jun is not necessary for the survival and development of SCs, it has long been know that c-jun is rapidly expressed at high levels in SCs of injured nerves (Jessen and Mirsky, 2016). Our analysis indicates that Faslg may be related to classical β-catenin, NF-κB, and caspase-3 regulatory pathways *in vitro* and thus affect SC function. Faslg may play an important role in the degeneration and repair processes after sciatic nerve injury in rats via the β-catenin, NF-κB, and caspase-3 signaling pathways. It is interesting that the p-ERK/ERK, p-c-jun/c-jun, c-Fos and N-Ras signaling pathways may also undergo changes following Faslg siRNA treatment or overexpression *in vivo*.

In summary, our data suggest that Faslg plays an important role in degeneration and regeneration after sciatic nerve injury. Our findings explain the underlying mechanisms of the effects of Faslg on nerve degeneration and/or regeneration. Our findings provide support for the future use of gene therapy, cell therapy, and other basic biological methods for the treatment of nerve damage.

## Significance Statement

WD is associated with a large number of gene expression changes. However, the effects of these up- or down-regulated genes are poorly understood. The aim of this study was to investigate the roles of Faslg in nerve injury, repair, and regeneration in WD. The data indicated that Faslg expressions was up-regulated after sciatic nerve injury in rats. Altered expression of Faslg in SCs and the injured sciatic nerve affected SC proliferation, migration and apoptosis, as well as the expression of related factors and signaling pathways *in vivo* and *in vitro*. This study sheds new light on the role of Faslg in peripheral nerve degeneration and/or regeneration.

## Author Contributions

YL and YS performed the experiments. MC was responsible for gene expression analysis. HZ conducted the animal studies. NG performed data analysis. HH performed immunohistochemical experiments. SC analyzed the functional and biochemical data. DY planned the study and wrote the manuscript. All authors have approved the manuscript and provided consent for its publication.

## Conflict of Interest Statement

The authors declare that the research was conducted in the absence of any commercial or financial relationships that could be construed as a potential conflict of interest.

## Abbreviations

Bax: Bcl-associated X protein
bcl-2: B-cell lymphoma-2
bFGF: basic fibroblast growth factor
CNS: central nervous system
EdU: 5-Ethynyl-2′-deoxyuridine
EGF: epidermal growth factor
ERK: extracellular regulated protein kinase
Faslg: Fas Ligand Gene
ICC: immunocytochemistry
JaK: Janus kinase
JNK: c-Jun NH2-terminal kinase
MMP: matrix metallopeptidase
NC: the negative control that transfected with the non-specific sequence
NF2: neurofibromin 2
NF-κB: nuclear factor-κB
N-Ras: neuroblastoma Ras
NT3: Neurotrophin 3
PKCα: protein kinase C α
qPCR: quantitative polymerase chain reaction
SC: Schwann cell
SD: Sprague-Dawley
SEM: standard error of the mean
siRNA: small interfering RNA
TUNEL: terminal deoxynucleotidyl transferase-mediated nick-end labeling
WD: Wallerian degeneration.
